# Body Dissatisfaction and Body-Related Attentional Bias: Is There a Causal Relationship?

**DOI:** 10.3390/jcm12175659

**Published:** 2023-08-30

**Authors:** María Teresa Mendoza-Medialdea, Franck-Alexandre Meschberger-Annweiler, Mariarca Ascione, Alejandra Rueda-Pina, Elisa Rabarbari, Bruno Porras-Garcia, Marta Ferrer-Garcia, José Gutiérrez-Maldonado

**Affiliations:** 1 Department of Psychology, Universidad de Jaén, Paraje las Lagunillas s/n, 23071 Jaén, Spain; tmedoza@ub.edu; 2 Department of Clinical Psychology and Psychobiology, Faculty of Psychology, University of Barcelona, Passeig de Vall d’Hebron 175, 08035 Barcelona, Spaine.rabarbari@gmail.com (E.R.);; 3 Brain, Cognition, and Behavior Research Group, Consorci Sanitari de Terrassa (CST), Ctr. Torrebonica, s/n, 08227 Terrassa, Spain; 4 Department of Basic Sciences, Universitat Internacional de Catalunya, Josep Trueta Street, s/n, 08195 Sant Cugat del Vallès, Spain

**Keywords:** body dissatisfaction, attentional bias, eye tracking, virtual reality, avoidance

## Abstract

Previous research has shown an association between body dissatisfaction and attentional biases toward the body, but the nature of this relationship is not clear. It is possible that dissatisfaction causes attentional bias or that dissatisfaction is a result of such bias. To clarify the causal relationship between these two variables, this study manipulated dissatisfaction in a sample of healthy women by exposing them to images of “ideal” bodies and observed whether this manipulation increased attentional biases toward different body parts. Fifty-seven women took part in a pre–post experimental design in which they observed an avatar representing themselves in a virtual mirror before and after being exposed to “thin ideal” photographs. Eye-tracking technology was employed to quantify the frequency and duration of fixations on weight-related and weight-unrelated body parts. The outcomes revealed a successful induction of body dissatisfaction, leading participants to display a heightened number of fixations and prolonged fixation durations on unrelated-weight body parts. These findings remained significant after controlling for the effects of trait body dissatisfaction and body mass index. The results imply that heightened body dissatisfaction fosters the aversion of attention from weight-related body parts, which may function as a protective mechanism for preserving self-esteem and promoting psychological well-being.

## 1. Introduction

Previous research has demonstrated a significant association between body dissatisfaction and attentional biases directed toward the body [1]. However, the precise nature of the relationship between body dissatisfaction and attentional biases remains unclear. Two possible explanations for this association can be proposed. Firstly, it is plausible that body dissatisfaction may lead to the development of attentional biases, wherein individuals allocate their attention more readily to stimuli (or avoid them) that confirm or reinforce their negative body image. In this scenario, individuals with higher levels of dissatisfaction may exhibit heightened sensitivity to appearance-related cues, directing their attention toward perceived flaws or discrepancies in their own bodies or actively avoiding them. This biased attentional focus may intensify their feelings of dissatisfaction, creating a self-perpetuating cycle. Alternatively, it is also possible that attentional biases toward specific body-related stimuli contribute to the development or exacerbation of body dissatisfaction. Under this perspective, individuals who exhibit heightened attentional biases toward appearance-related cues may be more likely to notice and internalize societal beauty ideals, leading to increased levels of body dissatisfaction. Consequently, this attentional bias may contribute to the perpetuation and reinforcement of negative body image perceptions.

The “thin ideal” is a social norm that defines beauty as having a slim and toned body, regardless of other factors such as health, personality, or diversity, making it unattainable for most women. This standard has been strengthened and disseminated by mass media, including magazines and television, which often feature models and celebrities who conform to this ideal or have been digitally manipulated to appear thinner and more attractive. In recent years, the proliferation and internalization of the “thin ideal” have been amplified by social media platforms [2,3]. These platforms allow users to share and manipulate their own images and videos, as well as engage in comparisons with others [4,5], thereby fostering the spread and acceptance of this ideal.

Exposure to idealized body images in the media has been shown to have a detrimental effect on women’s body image [6,7,8]. The disparity between a woman’s actual appearance and the perceived ideal can result in significant feelings of body dissatisfaction [2,9]. Body dissatisfaction is a prevalent and significant issue that impacts women across various age groups and backgrounds [10,11], with prevalence rates ranging from 11% to 30% among young Western women [12]. It serves as both a risk and maintenance factor for eating disorders [13]. Furthermore, body dissatisfaction also moderates the negative effects of other factors, such as low self-esteem or depression [14,15].

A related phenomenon observed among women with high levels of body dissatisfaction is the presence of an attentional bias toward images associated with thinness. These individuals struggle to redirect their attention away from thin bodies [16,17] and exhibit heightened attention toward such stimuli [18]. This phenomenon influences and perpetuates body image disturbances [19]. Studies employing eye-tracking (ET) methodologies provide further evidence of this attentional bias [1,19]. These studies offer continuous measures of attention over time, demonstrating longer fixation durations and a greater number of fixations on specific stimuli [20], as well as longer latencies and durations of the first fixation [21].

Various attentional patterns have been identified in experimental eye-tracking studies that incorporate participants’ own bodies as stimuli. The diversity of methods used to assess attentional biases may contribute to these differing findings. For example, some studies used a photograph of the participant’s body that can be anonymized (blurred head or without head) or not [22,23]; others presented a photograph of the most attractive or unattractive body parts of the participants [24]; some studies introduced changes in the body size of the participant’s avatar [25]. Women with eating disorders tend to direct their attention toward body parts they perceive as more dissatisfying or unattractive, with this bias being more pronounced among those with higher levels of body dissatisfaction (see [26] for a review). Similar attentional patterns have been observed among women without eating disorders [25,27] and those with high levels of body dissatisfaction [28]. However, contrasting results have been reported in other studies, indicating an avoidance of the most dissatisfied or unattractive self-body parts [29]. Additionally, obese women with higher levels of body dissatisfaction tend to exhibit a bias toward their self-perceived attractive body parts [23]. When comparing women with high and low levels of body dissatisfaction, it has been observed that those with high body dissatisfaction spend less time observing the self-body parts they are dissatisfied with compared to women with low body dissatisfaction [22].

However, these previous studies primarily focus on women who already exhibit a certain level of body dissatisfaction, neglecting to explore the temporal progression of increasing body dissatisfaction, which more closely reflects real-life experiences when exposed to mass media portrayals of the “thin ideal”. A commonly employed method to study body dissatisfaction involves inducing it through photographs of women who embody the “thin ideal”. A brief period of exposure (10 min or less) can result in an increase in body dissatisfaction [6]. However, these studies primarily focus on monitoring changes in body satisfaction or related subjective variables rather than investigating attentional biases or their role in the development and maintenance of body dissatisfaction.

To investigate the causal nature of the connection between body dissatisfaction and attentional biases, it is crucial to utilize experimental methodology. This study aims to investigate how the induction of body dissatisfaction affects the attentional biases displayed by healthy women when observing an avatar representing their own bodies in a virtual mirror that examines whether dissatisfaction can be considered a causal factor in attentional biases toward the body. This will be achieved by inducing body dissatisfaction in a group of healthy women through exposure to photographs portraying the “thin ideal”. Participants’ attentional bias will be assessed using ET technology before and after the exposure to the photographs. In order to further explore the effects, participants’ body mass index (BMI) and body dissatisfaction traits will be controlled for. Previous research has indicated that individuals’ BMI can moderate the effects of body dissatisfaction and/or attentional bias [18,19,30]. Controlling for participants’ body dissatisfaction traits aims to ensure that their pre-existing level of body dissatisfaction does not impact the induction of body dissatisfaction. Based on previous evidence, it is anticipated that an increase in body dissatisfaction among women will result in some form of attentional bias toward their bodies. This study has the potential to contribute to a better understanding of the psychological mechanisms underlying body dissatisfaction and its consequences.

## 2. Materials and Methods

### 2.1. Participants

Fifty-nine college women from the University of Barcelona voluntarily participated in this study. All participants were recruited through social networks. Exclusion criteria were a self-reported diagnosis of a current eating disorder, a current self-reported severe mental disorder (e.g., schizophrenia or bipolar disorder), epilepsy, or a high level of astigmatism. Two participants were excluded due to device malfunction. The final sample consisted of 57 women, with a mean age of 24.54 years (SD = 3.71, range: 21–40) and a mean BMI (kg/m^2^) of 22.34 (SD = 3.32, range: 18–34).

### 2.2. Stimuli, Materials, and Procedure

#### 2.2.1. Hardware and Software

The device used to immerse the participants in the virtual reality (VR) scenario was a head-mounted display (HMD), HTC VIVE Pro Eye™ (HTC Corporation, Taoyuan, Taiwan), which included dual-OLED displays with a combined resolution of 2880 × 1600 pixels and 615 PPI. To track the movements of the participants and apply them to the avatar in real-time, five body trackers were used: the HMD, two VR controllers that the participants had to hold in their hands, and two feet trackers (VIVE trackers V3.0; HTC Corporation, Taoyuan, Taiwan); all devices communicated wirelessly with four SteamVR Base Station 2.0™ (Valve Corporation, Bellevue, WA, USA). The four base stations created a play area (up to 10 × 10 m) with high stability and precise tracking. The HMD HTC VIVE Pro Eye was equipped with Tobii™ eye-tracking technology (Tobii Technology, Stockholm, Sweden), enabling highly accurate detection of eye movements (binocular gaze data output frequency: 120 Hz, spatial accuracy ranging between 0.5 and 1.1 degrees, and a 5-point calibration process).

The VR environment was developed using Unity 3D v. 5.6.1 software (Unity Technologies, San Francisco, CA, USA), and the avatar was designed using Blender v. 2.78 software (Blender Foundation, Amsterdam, The Netherlands). The environment consisted of a room devoid of furniture, except for a large mirror positioned 1.54 m in front of the participant’s avatar. Additionally, two boxes were placed on the floor beside the participant. The mirror displayed a full-body image of the participant’s avatar and accurately mirrored all the movements performed by the participant. The avatar was constructed using two participant photos that faithfully captured the participant’s silhouette. The avatar’s skin tone could be adjusted to match the participant’s own complexion. The avatars were attired in t-shirts and trousers, with their colors customizable to match the actual clothing colors of the participants. They also sported shoes, HMD glasses, and grey caps. These latter two accessories were utilized to minimize disparities between the participant’s facial features and hairstyle and those of the avatar, as these characteristics were unalterable within the VR environment.

#### 2.2.2. Assessment Measures

Body satisfaction. The Spanish Body Image State Scale (SBISS [31]; Spanish version: [32]) was utilized to assess momentary body image satisfaction. It consisted of 6 items measured on a 9-point Likert-type scale. An example of the items used is: “Right now I feel (extremely dissatisfied; mostly dissatisfied; moderately dissatisfied; slightly dissatisfied; neither dissatisfied nor satisfied; slightly satisfied; moderately satisfied; mostly satisfied; extremely satisfied) with my physical appearance”. The Cronbach’s alpha for the Spanish version is 0.92. In this study, the Cronbach’s alpha was 0.88 during the pre-induction phase and 0.84 during the post-induction phase. Additionally, momentary body satisfaction was evaluated within the virtual reality (VR) environment using a Visual Analog Scale (VAS) ranging from 0 (not at all) to 100 (completely) with the question “Please indicate to what extent you feel satisfied at this moment with the appearance of your body”.

Fear of gaining weight was also assessed within the VR environment using a VAS ranging from 0 (not at all) to 100 (completely) with the question, “Please indicate to what extent you would be afraid of gaining weight at this time”.

Body dissatisfaction as a trait was evaluated using the Eating Disorder Inventory 3—Body Dissatisfaction questionnaire (EDI-BD [33]; Spanish version: [34]). The EDI-BD assesses the negative subjective attitude or evaluation of one’s body and specific body areas. It consisted of 10 items measured on a 6-point Likert-type scale, ranging from 0 (never) to 5 (always). An example of the items used is: “I think that my thighs are too big”. The Cronbach’s alpha for the Spanish version ranged from 0.67 to 0.9. In this study, the Cronbach’s alpha was 0.86.

#### 2.2.3. Body-Related Attentional Measures

To assess the attentional bias toward various body parts, participants were instructed to assume a position with slightly separated arms and legs. While maintaining this position, they were asked to gaze at their own reflection in the virtual mirror for a duration of 30 s. This procedure has been utilized in prior studies (e.g., [35,36]). The eye-tracking (ET) data was imported into the OGAMA v. 5.1 software (Freie Universität, Berlin, Germany) to extract fixation information both before and after the intervention that induced dissatisfaction.

Attentional measures, namely the number of fixations (NF) and complete fixation time (CFT), were evaluated based on the participants’ visual fixation on their own bodies, represented by the avatar in the virtual mirror. Visual fixation is defined as the act of maintaining a gaze on specific body-related areas of interest (AOIs) for a minimum duration of 100 milliseconds [37]. CFT was calculated by summing the fixation time (in milliseconds) across specific AOI groups, while NF was estimated by summing the total number of fixations on specific AOI groups. Both measures have been utilized in previous studies utilizing ET technologies [26] and are considered reliable for providing a continuous assessment of visual attention.

Two sets of AOIs were established based on the PASTAS questionnaire [38]. The PASTAS is a self-reported questionnaire that assesses body anxiety toward specific parts of the body. The weight-related AOIs (W-AOI) encompassed the waist, stomach, hips, thighs, and legs. The non-weight-related AOIs (NW-AOI) comprised the neck, shoulders, arms, chest, and feet. The participant’s head was excluded from both groups due to the presence of VR glasses worn by both the participant and the avatar. The VR glasses had the potential to capture more attention than the participant’s own head.

To compute the attentional bias measures NF and CFT, the total scores of NW-AOI were subtracted from the total scores of W-AOI. Consequently, both measures could yield positive or negative values, depending on whether the participant’s attention was predominantly focused on W-AOI (positive values) or NW-AOI (negative values).

#### 2.2.4. Body Dissatisfaction Induction Images

Fifteen images depicting idealized women were utilized to induce body dissatisfaction. Previous research has demonstrated the effectiveness of such images in inducing temporary body dissatisfaction (see [6] for a review). These images were sourced from public Instagram accounts and freely available resources on the internet, specifically from platforms like Pexels. All photographs were in color and featured full-body shots of slim and toned women without signs of a slight belly or sagging breasts. The women in the images were depicted wearing swimsuits, underwear, or sportswear that clearly highlighted their body shape. To ensure the reliability and consistency of the image selection process, three independent researchers individually evaluated a diverse range of images. Each researcher applied their own subjective criteria to identify images that represented the “thin ideal body”. Subsequently, the selections made by the three researchers were compared, and a final set of images was determined.

#### 2.2.5. Procedure

**Figure 1:**
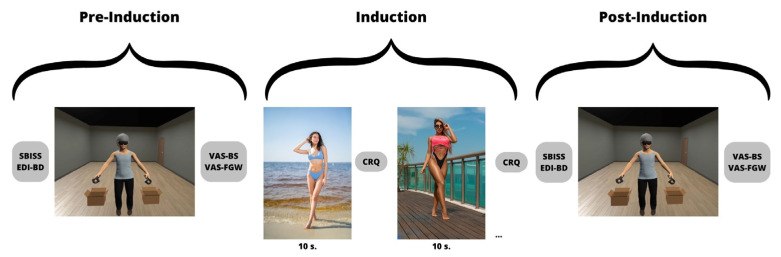
Experimental design scheme. SBISS = Spanish Body Image State Scale; EDI-BD = Eating Disorder Inventory—Body Dissatisfaction questionnaire; AB = attentional bias; VAS-BS = Visual Analog Scale Body Satisfaction; VAS-FGW = Visual Analog Scale Fear of Gaining Weight; CRQ = Consumer Response Questionnaire.

The Research Ethics Committee of the University of Barcelona approved the experimental protocol of this study, and written informed consent was obtained from all participants prior to the study. The researcher provided a cover story to the participants regarding the nature of this study in order to avoid interfering with the results. They explained that this study focused on body image on Instagram and involved a sustained attention task in VR. Once the session was completed, the participants were given accurate information regarding the true purpose of this study. The researcher explored by interview the presence of exclusion criteria and measured the weight and height of the participants to calculate their BMI. Two photos (frontal and lateral) were taken to create the participant’s avatar that accurately represented the participant’s silhouette. Figure 1 shows the general procedure of the experiment, with the appearance of the participant’s avatar, as well as an example of the photographs used during the induction of body dissatisfaction.

Prior to undergoing the body dissatisfaction induction procedure, the participant was requested to complete the assessment of momentary and trait body satisfaction using the SBISS and EDI-BD measures. Then, the participant was equipped with the HTC VIVE Pro Eye, two hand controllers, and two foot trackers and was immersed in the VR room. Next, a visuo-motor and a visuo-tactile stimulation procedure was applied to elicit a full body ownership illusion. This procedure is used to enhance the feeling that the avatar reflects the participant’s real body [36,39,40]. Next, the participant was instructed to look at themselves in the mirror for a duration of 30 s. The resulting pattern of fixations was recorded as a measure of visual attentional behavior prior to the induction of body dissatisfaction. Following that, the VAS questions were administered, and subsequently, the VR devices were removed.

Then, the induction of body dissatisfaction was introduced. A series of images depicting idealized women were presented on a computer screen for 10 s each. After each image, the participant completed the adapted Consumer Response Questionnaire [41], responding to statements such as “I would like my body to look like this woman’s body”, “This woman is thinner than me”, and “I would not like to try on a swimsuit if this woman was also trying on a swimsuit in the same changing room”. The questionnaire utilized a 5-point Likert scale ranging from 1 (strongly disagree) to 5 (strongly agree). The purpose was to increase body dissatisfaction by encouraging participants to directly compare themselves with the “ideal women”, a strategy known to be effective [7,42]. Once again, the VR devices were placed on the participant, and the visual behavior was recorded for another 30 s. Subsequently, the VAS questions were administered again, and after that, all VR devices were removed. In the final part of the experiment, the participant completed the body satisfaction questionnaire (SBISS) once more. Finally, the researcher explained the true objective of the study and addressed any questions from the participants.

#### 2.2.6. Statistical Analyses

For the statistical analysis of the self-reported data, the paired sample *t*-test was carried out to compare the total scores of each questionnaire before and after the induction of body dissatisfaction. The sample size for the analysis of VAS measures was 54 participants due to a failure to record the responses of three participants. For the attentional measures, two repeated measures ANCOVAs were performed to compare pre- and post-intervention scores, controlling for participants’ BMI and trait body dissatisfaction (EDI-BD) as covariates. The Kolmogorov–Smirnov test showed that the data were not normally distributed in all variables. However, it was decided to conduct the analyses since the ANCOVA has shown to be robust in case of deviation from normality. No outliers were detected in any variable. Effect sizes were also reported using the *r* method for the *t*-test and the partial ƞ^2^ for the ANCOVAs. The critical level of statistical significance was set at *α* = 0.05.

## 3. Results

### 3.1. Self-Reported Data

Table 1 indicates the data obtained from the self-reported questionnaires administered before and after the intervention, including the SBISS, the VAS of Body Satisfaction (VAS-BS), the VAS of Fear of Gaining Weight (VAS-FGW), and the EDI-BD.

According to the EDI-BD ratings for non-clinical populations [34], prior to the intervention, only two participants expressed dissatisfaction with their overall body shape and specific body parts, but not to the extent typically seen in eating disorders [34]. The remaining participants did not report any body dissatisfaction, scoring below the threshold established by the EDI-BD.

The analysis of the self-reported data indicated a statistically significant difference between the pre- and post-induction assessments for the SBISS and VAS-BS measures. Specifically, the SBISS scores indicated a significant decrease in body satisfaction following the intervention: *t*(53) = 2.879, *p* = 0.006, r = 0.135. Similarly, the VAS-BS scores showed a significant decrease in body satisfaction: *t*(56) = 2.411, *p* = 0.019, r = 0.095. No significant differences were found in the VAS-FGW scores between the pre- and post-induction assessments: *t*(53) = −0.92, *p* = 0.927, r = 0.018.

### 3.2. Body-Related Attentional Measures

The one-way ANCOVA results showed statistically significant differences between pre- and post-induction on the two attentional measures: the complete fixation time (*F*(1,54) = 10.776, *p* = 0.002, partial η^2^ = 0.166) and the number of fixations (*F*(1,54) = 22.511, *p* < 0.001, partial η^2^ = 0.294) after controlling for BMI and EDI-BD. As shown in Table 1, compared to the measures obtained before the induction of dissatisfaction, participants spent more time and had a greater number of fixations on non-weight-related areas during the post-induction assessment.

## 4. Discussion

The objective of this study was to investigate the impact of inducing body dissatisfaction on attentional biases in women while viewing an avatar representing themselves in a virtual mirror. The results demonstrated the effectiveness of inducing body dissatisfaction through exposure to photographs of women with an “ideal” thin body, as it led to a decrease in participants’ body satisfaction. Furthermore, as a result of increased body dissatisfaction, an attentional bias was observed in the participants, specifically toward non-weight-related body parts. This was indicated by an increased number of fixations and longer fixation durations in those areas. This effect remained significant even after controlling for participants’ BMI and trait body dissatisfaction, suggesting that attentional biases were specifically influenced by increased body dissatisfaction rather than participants’ current body size or their trait of body dissatisfaction [19,30].

These results substantiate the link between body dissatisfaction and the emergence of attentional biases. The escalation in body dissatisfaction corresponds to heightened sensitivity toward appearance-related cues, resulting in a deliberate avoidance of focusing attention on areas perceived as most flawed. Nevertheless, it is important to acknowledge that the connection between body dissatisfaction and attentional biases is not a simple one, and it is possible that this relationship could be reciprocal. Therefore, an attentional bias could affect the level of body dissatisfaction [24,43], creating a complex interplay between body dissatisfaction and attentional bias.

Similar results have already been reported in previous studies that used the participant’s own body as a stimulus. For instance, Janelle et al. [22] compared women with high and low body dissatisfaction viewing photographs of an aesthetic model as well as images of themselves while ET was recording. They found that the high body dissatisfaction group spent less time on weight-related areas in comparison to the low body dissatisfaction group. Similarly, von Wietersheim [29] employed 24 photographs of women with different BMIs, including a photograph of the participant, to monitor eye movements. They observed a tendency among individuals with anorexia nervosa to allocate less time and fewer fixations toward body parts that they perceived as negative. Warschburger et al. [23] also investigated the effects of body weight on visual attention using photographs of the participants and other persons divided into overweight and normal-weight groups. They found that the overweight group displayed a significantly longer fixation duration on attractive areas of their bodies compared with the normal-weight group. The results of these three studies suggest that the attentional bias toward non-weight-related body parts observed in our study may reflect an avoidance pattern. Previous research has suggested that a positive bias toward attractive body parts may have a protective effect, contributing to individuals’ mental health and well-being [27,44]. In our study, the sudden increase in body dissatisfaction may have triggered a regulatory mechanism aimed at returning body dissatisfaction to a baseline level by directing attention toward non-weight-related areas that are less discrepant with the “thin ideal” exposed here. This finding is consistent with previous studies that have found that women with high body dissatisfaction with the whole body or with specific body parts and women with a high drive for thinness tend to pay less attention to thin bodies of other women [45,46,47]. Specifically, Lykins et al. [45] found that dissatisfaction with specific body regions predicted avoidance of these regions when the participants looked at idealized models but also at plus-sized models, suggesting an avoidance of comparisons with others, whether detrimental or beneficial.

According to previous research, focusing on the most attractive body parts can improve body satisfaction. For instance, Smeets et al. [48] conducted a study on women with high body dissatisfaction, dividing them into two groups. One group viewed photographs of their three most attractive body parts, while the control group viewed photographs of all their body parts. The participants who focused only on their most attractive body parts increased body satisfaction in comparison to the control group, which showed no changes. A similar finding was observed by Jansen et al. [24], who asked two groups of females to look at their three most unattractive or attractive body parts, respectively, during three sessions. Both methods were effective in increasing body satisfaction. However, in the first two sessions of the group focusing on their unattractive body parts, body satisfaction decreased. In the third session, it increased similarly to the other group. Considering these two studies, focusing on the most attractive body parts may lead to a quicker increase in body satisfaction, although exposure to the most unpleasant areas can also produce positive change in the end. Nevertheless, it is first necessary to go through an initial increase in body dissatisfaction, which may temporarily distance individuals from their usual baseline.

The attentional bias observed in our study may provide insights into why a large portion of the population reports body dissatisfaction without developing eating disorders. People with eating disorders tend to allocate their attention to the most discomforting body areas of their body, perpetuating negative body image and potentially reinforcing maladaptive behaviors [19,26]. In contrast, the avoidance of weight-related areas in a healthy population could serve as a protective mechanism, preventing a decline in body satisfaction and mitigating the occurrence of negative thoughts and behaviors.

As mentioned in the introduction, other studies have reported contrasting results, suggesting that higher dissatisfaction is associated with increased attention toward body areas considered unattractive [19]. This discrepancy may stem from various factors or reasons. First, our study measured the state of body satisfaction before and after a body dissatisfaction induction in healthy women, whereas many previous studies examined individuals with eating disorders or high body dissatisfaction. Our participants in this study are likely more comparable to control or healthy groups that have been utilized in previous studies. These groups have shown a tendency to focus on their most attractive body parts or distribute their attention evenly across all body parts [26,35,44,49]. Based on our findings, it is possible that these control or healthy groups might exhibit an avoidance pattern similar to our sample if higher levels of body dissatisfaction were induced. However, further investigation is required to explore this possibility in more depth. Another difference is that previous studies have often examined the body as a whole without specifically investigating the potential differential effects of individual body parts on gaze patterns, particularly in relation to their weight-related or non-weight-related characteristics [35,36]. In our study, we took into account the influence of different body parts separately, which revealed distinct gaze patterns depending on whether the areas were weight-related or not.

This study has some limitations that should be considered. Future lines of research will also be proposed. First, this study was conducted only with healthy women, neglecting the growing issue of body dissatisfaction in men, who are pressured to have a “muscular ideal” physique [19], or patients with anorexia nervosa, who tend to allocate the attention to more unpleasant areas of their bodies [19]. Future research should consider including male participants and individuals with eating disorders to examine whether similar attentional biases and avoidance patterns exist in these populations. Another limitation of this study is that during the eye tracking, participants were required to maintain a fixed position, which restricted their ability to observe the back of their body, specifically the area where the buttocks are located, which is often associated with weight and beauty standards [50]. Including a setup that allows participants to view their entire body, potentially through the use of additional mirrors or alternative techniques, would provide a more comprehensive and ecologically valid experience. Furthermore, the use of non-standardized photographs of “thin ideal” women is another limitation. Currently, there is no standardized database of photographs representing a specific ideal of beauty. The development of such tools would enhance the quality of research and facilitate better comparisons between studies. Additionally, the mass media has introduced diverse representations of ideal bodies, such as fitness bodies or more curvaceous figures, in recent years [5,51]. Future research could consider using different types of ideal body shapes that align with the subjective preferences of the participants.

In addition to these potential avenues for future research, suggestions to enhance the ecological validity of our controlled experimental work encompass integrating real-world contexts into the research design, conducting studies in environments that trigger body dissatisfaction like clothing stores, gyms, or social media platforms, which could offer more realistic insights into attentional biases. Moreover, delving into the effects of psychological interventions on attentional biases, such as mirror exposure therapies, and how they influence long-term body satisfaction and attentional patterns can provide a deeper understanding of the practical implications of our findings. Additionally, accounting for cultural and societal factors that shape beauty ideals and body dissatisfaction triggers across different cultures could illuminate the impact on attentional patterns. Leveraging online research platforms could further widen the participant pool, enabling the validation of our findings across diverse populations and contexts.

In summary, our study results provide evidence of a causal relationship between body dissatisfaction and attentional biases toward different body parts. Through the deliberate manipulation of dissatisfaction using idealized body images, we observed the emergence of an attentional bias toward non-weight-related body parts. Essentially, a temporary increase in body dissatisfaction can result in an avoidance response toward weight-related body parts when individuals perceive themselves in the mirror. This response pattern, which contrasts with what is typically observed in women with eating disorders or high levels of trait body dissatisfaction, may serve as a self-protective mechanism to preserve self-esteem and promote mental well-being. These findings underscore the potential for customized interventions aimed at addressing body dissatisfaction; by acknowledging the causal connection between body dissatisfaction and attentional biases, professionals can devise interventions that simultaneously target both aspects, potentially yielding more impactful results. Tailoring therapeutic strategies to harness the observed response pattern might contribute to improved outcomes for individuals affected by these conditions. Moreover, mirror exposure therapies and body acceptance strategies could be further developed in light of these outcomes.

## Figures and Tables

**Table 1:** Mean values (and standard deviations) obtained before and after the intervention by the participants in the different questionnaires and behavioral data.

	Pre-Induction	Post-Induction
SBISS	5.839 (1.423)	5.544 (1.359)
VAS-BS	66.806 (24.874)	61.528 (25.874)
VAS-FGW	45.88 (28.765)	46.065 (28.301)
EDI-BD	8.4 (7.141)	-
AB-CFT (ms)	12.25 (6223)	−717.47 (971)
AB-NF	−0.19 (12.39)	−1.07 (14.69)

Note: SBISS = Spanish Body Image State Scale; VAS-BS = Visual Analog Scale Body Satisfaction; VAS-FGW = Visual Analog Scale Fear of Gaining Weight; EDI-BD = Eating Disorder Inventory—Body Dissatisfaction questionnaire; AB-CFT = attentional bias complete fixation time; AB-NF = attentional bias number of fixations.

## Data Availability

The data presented in this study are available on request from the corresponding author.
